# Polymorphisms in genes controlling inflammation and tissue repair in rheumatoid arthritis: a case control study

**DOI:** 10.1186/1471-2350-12-36

**Published:** 2011-03-07

**Authors:** Marieke Emonts, Mieke JMW Hazes, Jeanine J Houwing-Duistermaat, Christa E van der Gaast-de Jongh, Lisette de Vogel, Huub KH Han, Jacques MGW Wouters, Jon D Laman, Radboud JEM Dolhain

**Affiliations:** 1 Department of Immunology, Erasmus MC, University Medical Center, Rotterdam, The Netherlands; 2 Department of Pediatric Infectious Diseases and Immunology, Erasmus MC-Sophia Children's Hospital, University Medical Center, Rotterdam, The Netherlands; Post box 2060, room Sk-2208, 3000 CB Rotterdam, The Netherlands; 3 Department of Rheumatology, Erasmus MC, University Medical Center, Rotterdam, The Netherlands; 4 Department of Medical Statistics, LUMC, Leiden University Medical Centre, Leiden, Netherlands; 5 Department of Pediatrics, Radboud University Nijmegen Medical Centre, Nijmegen, The Netherlands; 6 Department of Rheumatology, Maasstad Ziekenhuis, Rotterdam, The Netherlands; 7 Department of Rheumatology, Sint Franciscus Hospital, Rotterdam, The Netherlands

## Abstract

**Background:**

Various cytokines and inflammatory mediators are known to be involved in the pathogenesis of rheumatoid arthritis (RA). We hypothesized that polymorphisms in selected inflammatory response and tissue repair genes contribute to the susceptibility to and severity of RA.

**Methods:**

Polymorphisms in *TNFA*, *IL1B*, *IL4*, *IL6*, *IL8, IL10*, *PAI1*, *NOS2a*, *C1INH*, *PARP*, *TLR2 *and *TLR4 *were genotyped in 376 Caucasian RA patients and 463 healthy Caucasian controls using single base extension. Genotype distributions in patients were compared with those in controls. In addition, the association of polymorphisms with the need for anti-TNF-α treatment as a marker of RA severity was assessed.

**Results:**

The *IL8 *781 CC genotype was associated with early onset of disease. The *TNFA *-238 G/A polymorphism was differentially distributed between RA patients and controls, but only when not corrected for age and gender. None of the polymorphisms was associated with disease severity.

**Conclusions:**

We here report an association between *IL8 *781 C/T polymorphism and age of onset of RA. Our findings indicate that there might be a role for variations in genes involved in the immune response and in tissue repair in RA pathogenesis. Nevertheless, additional larger genomic and functional studies are required to further define their role in RA.

## Background

Rheumatoid arthritis (RA) is a severely disabling chronic inflammatory disease that affects millions of people worldwide. Like in other autoimmune diseases females are affected more often than males. The disease course differs widely between patients. While some have low grade disease which is easily controlled by therapy with one disease-modifying anti-rheumatic drug (DMARD), others suffer from rapidly progressive disease with erosions that is therapy resistant, resulting in the use of many different DMARDs or even the need for therapy with the new biologicals directed against TNF-α. Cytokines and other proteins involved in the inflammatory response play a role in RA. Pro-inflammatory cytokines such as TNF-α and IL-1β are regarded as key players in the pathogenesis of RA [1,2]. This is further underlined by the development of several anti-cytokine drugs in the treatment of RA, with anti-TNF monoclonal antibodies and soluble TNF-α receptor being the most effective. TNF-α is an important pro-inflammatory cytokine. IL-1β in its turn, stimulates expression of IL-6 and IL-8[2]. Since IL-8 is involved in the initiation and amplification of acute inflammatory responses and in chronic inflammation, genetic variations (indirectly) linked to alterations in expression could be associated to RA susceptibility or severity[3-5]. Antibodies directed against the IL-6 receptor are effective in the treatment of RA [6]. IL-4 and IL-10 on the other hand, have been suggested to ameliorate arthritis [7,8]. Deficiency of IL-4 is associated with increased severity of arthritis in a mouse model[9]. In addition, the V50 variant in the IL-4 receptor causing unresponsiveness to IL-4, is associated with rapidly erosive RA [10]. In addition to inflammatory tissue destruction, factors involved in tissue repair and necrosis or apoptosis are likely to be involved in erosive disease and may also codetermine disease susceptibility. Plasminogen activator inhibitor 1 (PAI-1) inhibits fibrinolysis and tissue repair [11,12]. Plasminogen deficiency is associated with decreased susceptibility to and severity of arthritis in mice models for RA [13]. Previously the influence of genetic polymorphisms in inflammatory diseases such as RA has become evident. The association with the HLA DRB1 locus is the best characterized and replicated [14,15]. Linkage studies in RA families and genome wide screening in case control studies have identified over 30 validated additional genetic loci associated with RA such as *HLADRB1*, *PTPN22*, *TNFAIP3*, *TRAF1*, *STAT4*, *CCR6*, *PXK*, 10p15,12q13 and 22q13 [14,16-23]. For a recent overview we refer to the review of Orozco and Barton [24]. We used the classic candidate gene approach, including immune response factors that had previously been shown to be involved in RA pathogenesis, or were likely to be involved. This approach increases the chance that when a SNP is found to be significantly associated with disease, it may actually be the functional SNP. However, the relatively small sample size does limit the chance of finding loci of modest effect size and confirmation in independent cohorts is required. The SNPs in the genes were selected on the basis of either a functional effect, both on transcription (e.g. *PAI1*, *IL6*, *IL10*, *IL4*, *IL1B*) and amino acid substitution, resulting in functional differences (e.g. *PARP*) [25-32]. In addition, polymorphisms, such as in *TNFA *and *IL8*, previously associated with RA or other inflammatory diseases were considered relevant as well, even if they were positioned in an intron [3,33-35].

Therefore, in this study including 376 RA patients and 463 controls, we investigated the possible association of polymorphisms in immune response genes (*TNFA*, *IL1B*, *IL4*, *IL6*, *IL8*, *IL10*, *C1INH*, *TLR2*, *TLR4*) and genes involved in tissue repair and apoptosis (*PAI1*, 5*NOS2a*, *PARP*) in relation to RA susceptibility, and with the use of anti-TNF-α therapy as selected marker of disease severity.

## Methods

### Participants

Non related Caucasian patients who met the 1987 ACR criteria for RA and visited the rheumatology outpatient clinic of the Erasmus MC university hospital (n = 76), the Medical Center Rijnmond Zuid (n = 195) or the St. Franciscus Gasthuis Hospital (n = 105), in 2005 were included in the study. Additionally, the patients had to have at least either a positive rheumatoid factor test, or a positive anti-CCP test, or joint erosions. The study was approved by the medical ethical boards of the Erasmus MC and participating hospitals: Medical Center Rijnmond Zuid and the St. Franciscus Gasthuis Hospital. Patients were included after written informed consent. Gender, age at inclusion in the study, age at diagnosis, clinical parameters and current and prior medication were derived from the clinical charts.

Since in The Netherlands treatment of RA is strictly aimed at achieving clinical remission, disease activity itself cannot be used as a marker for disease severity. For that reason it was chosen to use the need for therapy with anti-TNF as an alternative marker for disease severity. This can be done reliably since in the Netherlands the prescription of anti-TNF is strictly regulated and the costs for anti-TNF treatment are only reimbursed by the health insurance companies for RA-patients with therapy-resistant disease. Therapy-resistance is defined as failure of at least two disease-modifying anti-rheumatic drugs (DMARDs) including methotrexate, and still active disease (defined as DAS28 > 3.2) despite therapy with methotrexate 25 mg weekly or with methotrexate at the maximum tolerated dose. Blood was drawn for DNA isolation and routine laboratory analysis. Since the anti-CCP test has only recently been introduced into routine patient care, only for a minority of patients (n = 125) anti CCP status was known.

Caucasian controls representing the general adult population (n = 463) were derived from the Sanquin Blood Bank South West Region.

### Experimental procedures

#### DNA isolation

For RA patients DNA isolation from heparinized whole blood was performed as described previously [36]. DNA from controls was derived from whole blood with column methods using standard protocols (Qiagen, Leusden, The Netherlands).

#### Genotyping

Single base extension (SBE) analysis was used to determine genotypes of inducible nitric oxide synthase (*NOS2A*) S608L (rs2297518), poly (ADP-ribose) polymerase (*PARP*) V762A (rs1136410), complement component inhibitor-1 (*C1INH*) V480 M (rs4926), *PAI1 *-675 4G/5G (rs1799889), *IL4 *C-524T (rs2243250), *IL10 *G-1082A (rs1800896), *IL10 *C-819T (rs3021097), *IL1B *C-31T (rs1143627), *TNFA *A-863C (rs1800630), *TNFA *T-857C (rs1799724), *TNFA *G-376A (rs3093659), *TNFA *G-308A (rs1800629), *TNFA *G-238A (rs361525), *IL6 *G-174C (rs1800795), *IL8 *C781T intron (rs2227306), *TLR4 *D299G (rs4986790) and *TLR4 *T399I (rs4986791) http://www.ncbi.nlm.nih.gov/SNP/, Applied Biosystems, SNaPShot, standard protocol as supplied by the manufacturer, Nieuwerkerk aan den IJssel, The Netherlands). The genomic region of interest was amplified by PCR (Additional file 1: Table S1, S2, and S3). After purification, a single base extension was performed using a primer ending one nucleotide prior to the single nucleotide polymorphism (SNP) location. Both forward and reverse strands were tested. In a multiplex assay one of the primers was used to genotype the SNP of interest. Up to seven SNPs were analysed in one assay. A poly-T-tail attached to the primer combined with the use of a Liz size marker served to distinguish SNPs in the multiplex analysis (Additional file 1: Table S4). A subset of PCR samples was sequenced to confirm genotypes. All genotypes were annotated independently by two investigators who were blinded for the clinical data.

### Statistical analysis

Statistical analysis was performed using SPSS 11.5. Haplotype analysis was carried out with Thesias version 2 [37,38]. Genotype frequencies were compared between patients with RA and controls using the Cochrane-Armitage trend test for additive effects. The median number of different drugs used per year since the moment of diagnosis was assessed. Additionally, genotype distributions between patients who received anti-TNF-α medication were compared with those who did not. Binomial variables were analysed using Pearson's chi-square test (2df) or Fisher's exact test when appropriate. For continuous variables Student's T test or Mann-Whitney U test were used when appropriate. Verification of Hardy Weinberg equilibrium (HWE) of genotypes was performed using Chi-square test (1df). Probability (P) values < 0.05 were considered to be statistically significant. Significant probability values obtained were corrected for multiple testing using Bonferroni for the number SNPs tested (n = 17) and the number of tests performed (n = 3). Power calculations using Quanto revealed a power of 0.8 to detect an OR of 1.5 comparing patients and controls given an allele frequency of 0.1 and α of 0.05 [39].

## Results

### Patient characteristics

376 patients, mean age (sd) 59.3 years (13.7) and 463 controls mean age (sd) 39.1 years (8.5), were included in the study (p < 0.001). In the patient and control cohort 277 (73.7%) and 230 (49.7%) individuals were female, respectively (p < 0.001). All patients were included when comparing RA patients with healthy controls. For 6 patients records regarding medication use were incomplete due to (multiple) transfers of patients from other hospitals. For this reason the 370 patients for whom medication data was complete were included in the analysis regarding disease severity (Table 1).

**Table 1:** Patient characteristics

	Total	Treatment		
		
	RA patients n = 370	No anti-TNF n = 370	Anti-TNF n = 120	p
**Mean age (sd)**	59.1 (13.7)	60.8 (13.9)	55.7 (12.8)	0.001
**Female gender (%)**	272 (73.5)	177 (71)	95 (79)	0.10
**Median nr years with RA (min-max)**	8.5 (0-54)	8.7 (0-54)	8.3 (0-46)	0.13 (MW)
**Anti-CCP positive (%)^a^**	104/125 (83.2)	75/90 (83)	29/35 (83)	1.0
**Reumatoid factor positive (%)**	337/369 (91.3)	228 (91)	109/119 (92)	1.0
**Erosion (%)**	291/357 (81.5)	189/243 (78)	102/114 (90)	0.008
**Geometric mean number of DMARDs (95% CI)**	2.96 (2.79-3.13)	2.37 (2.22-2.52)	4.69 (4.41-4.99)	< 0.001
**Geometric mean DMARDs per disease year (95% CI)**	0.42 (0.37-0.47)	0.37 (0.31-0.43)	0.55 (0.47-0.64)	0.001
**Number of patients with current or past use of DMARDs (%)**				
Methotrexate	331 (89.5)	212 (85)	119 (99)	< 0.001
Salazopyrine	262 (70.8)	162 (65)	100 (83)	< 0.001
Antimalaria	176 (47.6)	105 (42)	71 (59)	0.003
Leflunomide	33 (8.9)	16 (6)	17 (14)	0.02
Intramuscular gold	55 (14.9)	36 (14)	19 (16)	0.76
Infliximab	35 (9.5)	0	35 (29)	< 0.001
Etanercept	61 (16.5)	0	61 (51)	< 0.001
Adalilumab	60 (16.2)	0	60 (50)	< 0.001
Anakinra	14 (3.8)	2 (1)	12 (10)	< 0.001
Oral corticosteroids	164 (44.3)	103 (41)	61 (51)	0.09
Azathioprine	33 (8.9)	13 (5)	20 (17)	0.001
Others				
n = 1	32 (8.6)	17 (6.8)	15 (12.5)	0.01
n = 2	5 (1.4)	1 (0.4)	4 (3.3)	

a Anti-CCP was not routinely analyzed.

MW: Mann Witney

### Genotypes and disease susceptibility

In controls, genotype distribution of all SNPs except the *IL10 *G-819A polymorphism reached Hardy Weinberg Equilibrium (HWE). To rule out a technical problem, an initial set of individuals was typed using both the reverse and forward primer in the single base extension reaction. Results for both strands were identical. Sequencing of a subset of 13 random controls showed identical genotypes excluding technical errors (data not shown). When Bonferroni correction is applied, none of the SNPs was significantly associated with RA susceptibility, adjusted for age and gender. Using the Cochrane-Armitage trend test for additive effects an association was observed for the *TNFA *-238 G/A polymorphisms (Bonferroni corrected p values 0.04). The TNFA-238 G/G genotype were overrepresented in RA patients when compared to controls (crude OR (95% CI) 2.48 (1.38-4.46) for *TNFA *G/G vs G/A; Table 2). Analysis of *TNFA *haplotypes yielded similar results as for the individual SNPs (data not shown). Since RA patients were significantly older than healthy controls and controls are by definition still 'at risk' to develop RA at a later age, the difference observed may also result from a difference in susceptibility to develop RA at a younger age. For this reason the relation of all SNPs with the age at diagnosis was analysed. The *IL8 *781 C/C genotype was associated with younger age at RA diagnosis compared to both the *IL8 *781 C/T and T/T genotype (mean age (SD): *IL8 *781 T/T 52.2 (14) years, T/C 50.0 (14) years and C/C 44.2 (13) years, respectively, p < 0.01, after Bonferroni correction; Table 3).

**Table 2:** Genotype frequencies in RA patients and controls

SNP	Genotype frequency		p trend^a^	SNP	Genotype frequency		p trend^a^
	Controls n (%)	RA patients n (%)			Controls n (%)	RA patients n (%)	
**IL6-174**				**NOS2a 608**			
C/C	82 (17.9)	56 (16.7)	0.01	G/G	301 (65.2)	254 (68.6)	0.22
C/G	231 (50.3)	133 (39.7)		G/A	140 (30.3)	104 (28.1)	
G/G	146 (31.8)	146 (43.6)		A/A	21 (4.5)	12 (3.2)	
**IL8 781**				**PARP 762**			
C/C	152 (32.9)	141 (37.5)	0.4	T/T	299 (66.6)	261 (70.7)	0.40
C/T	234 (50.6)	171 (45.5)		T/C	140 (31.2)	96 (26.0)	
T/T	76 (16.5)	64 (17.0)		C/C	10 (2.2)	12 (3.3)	
**PAI1 4/5G**				**IL4-524**			
5G/5G	99 (21.4)	101 (27.7)	0.12	C/C	339 (73.7)	267 (71.8)	0.30
5G/4G	240 (51.9)	172 (47.1)		C/T	112 (24.3)	91 (24.5)	
4G/4G	123 (26.6)	92 (25.2)		T/T	9 (2.0)	14 (3.8)	
**TNFA-863**				**C1INH 480**			
C/C	329 (71.4)	239 (64.8)	0.16	G/G	255 (55.4)	211 (58.3)	0.47
C/A	117 (25.4)	123 (33.3)		G/A	176 (38.3)	129 (35.6)	
A/A	15 (3.3)	7 (1.9)		A/A	29 (6.3)	22 (6.1)	
**TNFA-857**				**Il10-1082**			
C/C	386 (84.5)	314 (86.5)	0.29	G/G	123 (26.8)	88 (24.7)	0.77
C/T	66 (14.4)	48 (13.2)		G/A	219 (47.7)	172 (48.3)	
T/T	5 (1.1)	1 (0.3)		A/A	117 (25.5)	96 (27)	
**TNFA-376**				**IL10-819**			
G/G	446 (96.7)	371 (98.9)	0.04	C/C	283 (61.5)	233 (63.7)	0.99
G/A	15 (3.3)	4 (1.1)		C/T	145 (31.5)	100 (27.3)	
A/A				T/T	32 (7.0)	33 (9.0)	
**TNFA-308**				**TLR4 299**			
G/G	300 (65.1)	248 (66.1)	0.60	A/A	373 (86.3)	315 (85.1)	0.62
G/A	147 (31.9)	119 (31.7)		A/G	58 (13.4)	54 (14.6)	
A/A	14 (3.0)	8 (2.1)		G/G	1 (0.2)	1 (0.3)	
**TNFA-238**				**TLR4 399**			
G/G	415 (90.0)	359 (95.7)	0.002	C/C	378 (87.1)	314 (84.9)	0.42
G/A	46 (10.0)	16 (4.3)		C/T	54 (12.4)	55 (14.9)	
A/A				T/T	2 (0.5)	1 (0.3)	
**IL1B-31**							
C/C	57 (12.5)	48 (12.8)	0.62				
C/T	214 (46.9)	165 (44.0)					
T/T	185 (40.6)	162 (43.2)					

a Cochrane-Armitage trend test for additive effects; p value represents analysis not corrected for multiple testing. When Bonferroni correction is applied (factor 20 for the number of SNPs (17) and tests (3)) only the TNFA-238 polymorphism remains significant (p = 0.04).

**Table 3:** Age of onset of RA in relation to genotypes

SNP	Age of onset	p	SNP	Age of onset	p
	Mean (SD)			Mean (SD)	
**IL6-174**			**NOS2a 608**		
C/C	48 (11)	0.16	G/G	47 (13)	0.10
C/G	50 (15)		G/A	51 (15)	
G/G	47 (15)		A/A	49 (17)	
**IL8 781**			**PARP 762**		
C/C	44 (13)	< 0.0001	T/T	48 (14)	0.44
C/T	50 (14)		T/C	48 (15)	
T/T	52 (14)		C/C	53 (13)	
**PAI1 4/5G**			**IL4-524**		
5G/5G	46 (15)	0.22	C/C	48 (14)	0.48
5G/4G	49 (13)		C/T	50 (14)	
4G/4G	49 (14)		T/T	49 (16)	
**TNFA-863**			**C1INH 480**		
C/C	48 (14)	0.40	G/G	48 (14)	0.41
C/A	48 (13)		G/A	48 (15)	
A/A	55 (20)		A/A	52 (12)	
**TNFA-857**			**Il10-1082**		
C/C	49 (14)	0.75	G/G	49 (14)	0.90
C/T	48 (14)		G/A	48 (14)	
T/T	38 (-) (n = 1)		A/A	148 (14)	
**TNFA-376**			**IL10-819**		
G/G	48 (14)	0.01	C/C	49 (14)	0.10
G/A	30 (14)		C/T	46 (14)	
A/A			T/T	49 (14)	
**TNFA-308**			**TLR4 299**		
G/G	48 (14)	0.69	A/A	48 (14)	0.86
G/A	49 (14)		A/G	48 (13)	
A/A	52 (14)		G/G	56 (-)(n = 1)	
**TNFA-238**			**TLR4 399**		
G/G	48 (14)	0.40	C/C	48 (14)	0.82
G/A	45 (15)		C/T	49 (14)	
A/A			T/T	56 (-)(n = 1)	
**IL1B-31**					
C/C	47 (15)	0.84			
C/T	48 (15)				
T/T	48 (13)				

### Genetic polymorphisms and severity of disease

The use of TNF-α modifying therapy was considered to be an indicator of severe disease in the Dutch situation as explained in the methods section. Anti-TNF-α drugs were administered to 120 of 370 patients. The mean age (sd) of patients who used anti-TNF-α was 55.7 (12.8) years compared with 60.8 (13.9) years in the group that did not receive anti-TNF-α (p = 0.001). Presence of anti CCP antibodies, RF or disease duration was not different between the two groups. In contrast, the presence of erosions, and the numbers of DMARDs used was differentially distributed between patients requiring anti-TNF treatment to obtain acceptable disease activity and those who did not (Table 1). Only a trend was observed for the *TNFA *-308 G/A polymorphism using the Cochrane-Armitage trend test for additive effects (p = 0.04, without Bonferroni correction). After adjustment for age and multiple testing, none of the polymorphisms was significantly associated with disease severity (Table 4).

**Table 4:** Comparison of genotype frequencies between RA patients with or without anti-TNF therapy, as marker of disease severity

SNP	Genotype frequency		p trend^a^	SNP	Genotype frequency		p trend^a^
	No anti-TNF	Anti-TNF			No anti-TNF	Anti-TNF	
	250 n (%)	120 n (%)			250 n (%)	120 n (%)	
**IL6-174**				**NOS2a 608**			
C/C	33 (15.1)	22 (19.8)	0.06	G/G	175 (70.9)	75 (64.1)	0.18
C/G	83 (37.9)	49 (44.1)		G/A	65 (26.3)	37 (31.6)	
G/G	103 (47.0)	40 (36.0)		A/A	7 (2.8)	5 (4.3)	
**IL8 781**				**PARP 762**			
C/C	97 (38.8)	43 (35.8)	0.92	T/T	180 (73.2)	75 (64.1)	0.37
C/T	108 (43.2)	58 (48.3)		T/C	55 (22.4)	41 (35.0)	
T/T	45 (18.0)	19 (15.8)		C/C	11 (4.5)	1 (0.9)	
**PAI1 4/5G**				**IL4-524**			
5G/5G	67 (27.6)	33 (28.4)	0.97	C/C	177 (71.7)	87 (73.1)	0.62
5G/4G	116 (47.7)	53 (45.7)		C/T	60 (24.3)	29 (24.4)	
4G/4G	60 (24.7)	30 (25.9)		T/T	10 (4)	3 (2.5)	
**TNFA-863**				**C1INH 480**			
C/C	155 (62.8)	78 (67.2)	0.42	G/G	143 (58.8)	65 (57.5)	0.62
C/A	87 (35.2)	36 (31.0)		G/A	82 (33.7)	45 (39.8)	
A/A	5 (2.0)	2 (1.7)		A/A	18 (7.4)	3 (2.7)	
**TNFA-857**				**II10-1082**			
C/C	212 (87.2)	96 (84.2)	0.52	G/G	61 (25.7)	25 (21.9)	0.55
C/T	30 (12.3)	18 (15.8)		G/A	112 (47.3)	57 (50.0)	
T/T	1 (0.4)			A/A	64 (27.0)	32 (28.1)	
**TNFA-376**				**IL10-819**			
G/G	248 (99.2)	118 (98.3)	0.45	C/C	153 (63.2)	75 (63.6)	0.72
G/A	2 (0.8)	2 (1.7)		C/T	65 (26.9)	34 (28.8)	
A/A				T/T	24 (9.9)	9 (7.6)	
**TNFA-308**				**TLR4 299**			
G/G	156 (62.4)	89 (74.2)	0.13	A/A	206 (83.7)	104 (88.1)	0.24
G/A	91 (36.4)	26 (21.7)		A/G	39 (15.9)	14 (11.9)	
A/A	3 (1.2)	5 (4.2)		G/G	1 (0.4)		
**TNFA-238**				**TLR4 399**			
G/G	243 (97.2)	111 (92.5)	0.04	C/C	206 (83.7)	103 (87.3)	0.34
G/A	7 (2.8)	9 (7.5)		C/T	39 (15.9)	15 (12.7)	
A/A				T/T	1 (0.4)		
**IL1B-31**							
C/C	30 (12.0)	17 (14.3)	0.68				
C/T	116 (46.4)	46 (38.6)					
T/T	104 (41.6)	56 (47.1)					

Only patients for whom data regarding medication were complete (n = 370) were included.

a. Cochrane-Armitage trend test for additive effects. p value represents analysis not corrected for multiple testing. When Bonferroni correction is applied none of the polymorphism tested remain significant.

## Discussion

In our study the *IL8 *781 C/C genotype was associated with a younger age at RA diagnosis. Increased IL-8 expression was associated with RA when compared with osteoarthritis [40]. The *IL8 *C/T polymorphism was previously associated with viral infection, but no data are available on the effect of this intronic polymorphism on IL-8 expression. Recently a group in Taiwan published the IL-8 3'-UTR 2767AA genotype to be associated with younger age at onset [41]. The *IL8 *781C, *IL8 *276A and the *IL8 *-251T alleles appear to be in linkage disequilibrium [3]. The *IL8 *-251 T allele has previously been associated with respiratory syncytial virus infection in children, in whom also higher serum IL-8 levels were observed [3]. Given the role of IL-8 in promoting the pro-inflammatory response this could explain the association of *IL8 *polymorphisms with early onset of RA.

Only the *TNFA *-238 polymorphism was differentially distributed between RA patients and controls, even after Bonferroni correction. However, after adjustment for age and gender none remained significant. Using anti-TNF requirement as marker for severity a trend was observed for the *TNFA *-238 polymorphism. The findings for the TNFA-238 polymorphism match those reported for both a Mexican and a Colombian cohort [33,35]. Additionally, in the Mexican cohort, the -308 A allele was associated with more severe disease, while it is also reported to be associated with erosion in RA patients [35,42]. Moreover, carriage of the *TNFA *-308A allele, related to higher TNF-α production, was previously reported to be associated with non-response to anti-TNF-α in patients with different autoimmune diseases among which RA [43-45]. In the past numerous studies have been performed to investigate the association of *TNFA *promoter polymorphisms and TNF-α levels in different inflammatory and infectious diseases, reporting contradictory results [46]. TNF-α expression is probably not determined by one but by a combination of polymorphisms in *TNFA *and -associated genes. In our study, and in contrast to those described above, only the *TNFA *-238A allele showed a trend towards more severe disease (Table 3). It must however be noted that the allele frequency is very low and a slight difference in genotype distribution would markedly alter the results. Moreover, it is not unlikely that the observations for the *TNFA *polymorphisms result at least in part from linkage disequilibrium with the *HLADRB1 *locus, which was previously associated with RA [14,15,19].

No association of polymorphisms with disease severity was observed after correction for multiple testing. Many would argue that functional studies are preferred over genetic association studies. However, interpretation of RNA expression analyses is difficult due to the intra and inter-patient variation [47]. Careful sampling may overcome this problem and reduce the finding of false positive differentially expressed genes. With the exception of those studies assessing cytokine expression only in patients without DMARDs at diagnosis, results are likely to be influenced by the use of these DMARDs and disease progression. For this reason comparison of the results of different studies is limited. One can question whether therapy with anti-TNF is an appropriate marker for disease severity. In the modern era of aggressive treatment of RA medication is intensified till the goal of low disease activity or even remission is achieved. That disease activity was low is underscored by the low geometric mean ESR level in our cohort of RA-patients (16.2; 95% CI 14.7-17.7 mm/hr), that was not different (p = 0.17) between patients on conventional DMARDs (15.4; 95% CI 13.8-17.3 mm/hr) or those on biologicals (17.6; 95% CI 15.1-20.5 mm/hr). It was therefore decided not to use disease activity as marker of disease severity, but whether low disease activity could be achieved by conventional DMARDs or by use of biologicals directed against TNF. That therapy with a biological against TNF could be used as a marker of disease severity in this study is also due to the fact that its use is strictly regulated in the Netherlands; therapy with biologicals against TNF is available for all RA-patients that failed on two DMARDs and still have active disease (DAS28 > 3.2) despite therapy with methotrexate 25 mg weekly or at the maximal tolerated dose.

No association was observed for the *IL10 *polymorphisms with RA susceptibility or severity. Previously, contrasting results were reported for the role of IL10 polymorphisms in RA [48-50]. A recent study demonstrated a significant association between having RA and the IL10 1082 G allele (p = 0.008; OR = 1.44, 95% CI 1.11-1.86) [50]. Recently, a substantial improvement of RA upon treatment with anti-IL-6 receptor antibody [6]. The *IL6 *G/G genotype is associated with increased IL-6 levels compared to the C/C genotype, contributing to the complex regulation of IL-6 production [26,51-53]. Furthermore, IL-6 expression in synovial tissue is higher in end-stage RA than in chronic active RA [54]. IL-6 expression is influenced by TNF-α, and interaction of polymorphisms in these and other genes may co-determine the disease phenotype. This interaction in combination with the limited sample size may have prohibited finding an association of the IL6 polymorphism with RA.

Although a polymorphism in *TLR4 *has previously been associated with rapid response to treatment in RA no association with RA was observed for the *TLR4 *polymorphisms in our study [55].

Recent GWAS studies on RA susceptibility included thousands of patients. We used a candidate gene approach in the SNP selection because this is a moderately sized population. Despite having a larger a priori chance of finding a true association if SNPs are associated with disease, this may have resulted in both false positive and false negative findings. The recent studies observed no larger effect size than an OR of approximately 1.3. This implies that our study is probably underpowered to detect loci with a modest effect, and that larger patient numbers are needed to gain further insight into the possible role of these genes in RA pathogenesis. Additionally, apart from the *TNFA *region, the identified genomic regions of interest in these GWAS study do not correspond to the location of the genes we selected. Nevertheless, although validation of our results in an independent cohort is required, the data presented here do remain valuable for meta-analysis.

## Conclusions

In summary we here report an association between *IL8 *781 C/T polymorphism and a younger age of onset of RA. After correction for age (and gender), and multiple testing, none of the polymorphisms was associated with RA susceptibility or severity. This illustrates the need for additional larger studies to elucidate the contributing factor of polymorphisms in the susceptibility to and severity of RA. Carefully designed functional studies regarding expression of mRNA and protein levels in combination with genetic analyses are warranted to elucidate the contribution of polymorphisms to disease severity and will contribute to improving our knowledge on RA pathogenesis.

## Competing interests

The authors declare that they have no competing interests.

## Authors' contributions

ME and RD designed the study, got medical ethical approval, were responsible for database management and wrote the manuscript; JH and JL participated in the study design; ME and JH-D performed the statistical analyses. LV isolated the DNA; CG-J performed the genotyping analysis; KH, JW and RD included patients and collected clinical data. All authors read and approved the final manuscript.

## Pre-publication history

The pre-publication history for this paper can be accessed here:

http://www.biomedcentral.com/1471-2350/12/36/prepub

## Supplementary Material

Additional file 1 **Supplemental tables S1, S2, S3 and S4**. Supplemental table S1. PCR primer sequences. Supplemental table S2. PCR conditions using 384 Tetrad PCR machine. Supplemental table S3. Touchdown protocols for PCR. Supplemental table S4. Primer sequences for SBE reactions. Click here for file

## References

[B1] van den Berg WB Arguments for interleukin 1 as a target in chronic arthritis Ann Rheum Dis 2000 59 Suppl 1 i81 84 10.1136/ard.59.suppl_1.i81 11053095 PMC1766623

[B2] Zwerina J Redlich K Schett G Smolen JS Pathogenesis of rheumatoid arthritis: targeting cytokines Ann N Y Acad Sci 2005 1051 716 729 10.1196/annals.1361.116 16127012

[B3] Heinzmann A Ahlert I Kurz T Berner R Deichmann KA Association study suggests opposite effects of polymorphisms within IL8 on bronchial asthma and respiratory syncytial virus bronchiolitis J Allergy Clin Immunol 2004 114 671 676 10.1016/j.jaci.2004.06.038 15356575

[B4] Rodenburg RJ van Den Hoogen FH Barrera P van Venrooij WJ van De Putte LB Superinduction of interleukin 8 mRNA in activated monocyte derived macrophages from rheumatoid arthritis patients Ann Rheum Dis 1999 58 648 652 10.1136/ard.58.10.648 10491366 PMC1752770

[B5] Harada A Sekido N Akahoshi T Wada T Mukaida N Matsushima K Essential involvement of interleukin-8 (IL-8) in acute inflammation J Leukoc Biol 1994 56 559 564 7964163

[B6] Maini RN Taylor PC Szechinski J Pavelka K Broll J Balint G Emery P Raemen F Petersen J Smolen J Double-blind randomized controlled clinical trial of the interleukin-6 receptor antagonist, tocilizumab, in European patients with rheumatoid arthritis who had an incomplete response to methotrexate Arthritis Rheum 2006 54 2817 2829 10.1002/art.22033 16947782

[B7] Joosten LA Lubberts E Helsen MM Saxne T Coenen-de Roo CJ Heinegard D van den Berg WB Protection against cartilage and bone destruction by systemic interleukin-4 treatment in established murine type II collagen-induced arthritis Arthritis Res 1999 1 81 91 10.1186/ar14 11056663 PMC17779

[B8] Keystone E Wherry J Grint P IL-10 as a therapeutic strategy in the treatment of rheumatoid arthritis Rheum Dis Clin North Am 1998 24 629 639 10.1016/S0889-857X(05)70030-2 9710891

[B9] Finnegan A Grusby MJ Kaplan CD O'Neill SK Eibel H Koreny T Czipri M Mikecz K Zhang J IL-4 and IL-12 regulate proteoglycan-induced arthritis through Stat-dependent mechanisms J Immunol 2002 169 3345 3352 12218156 10.4049/jimmunol.169.6.3345

[B10] Prots I Skapenko A Wendler J Mattyasovszky S Yone CL Spriewald B Burkhardt H Rau R Kalden JR Lipsky PE Schulze-Koops H Association of the IL4R single-nucleotide polymorphism I50V with rapidly erosive rheumatoid arthritis Arthritis Rheum 2006 54 1491 1500 10.1002/art.21832 16646030

[B11] Kjoller L Kanse SM Kirkegaard T Rodenburg KW Ronne E Goodman SL Preissner KT Ossowski L Andreasen PA Plasminogen activator inhibitor-1 represses integrin- and vitronectin-mediated cell migration independently of its function as an inhibitor of plasminogen activation Exp Cell Res 1997 232 420 429 10.1006/excr.1997.3540 9168821

[B12] Stefansson S Lawrence DA The serpin PAI-1 inhibits cell migration by blocking integrin alpha V beta 3 binding to vitronectin Nature 1996 383 441 443 10.1038/383441a0 8837777

[B13] Li J Guo Y Holmdahl R Ny T Contrasting roles of plasminogen deficiency in different rheumatoid arthritis models Arthritis Rheum 2005 52 2541 2548 10.1002/art.21229 16052596

[B14] Criswell LA Gregersen PK Current understanding of the genetic aetiology of rheumatoid arthritis and likely future developments Rheumatology (Oxford) 2005 44 Suppl 4 iv9 iv13 10.1093/rheumatology/kei054 16306485

[B15] Gibert M Balandraud N Touinssi M Mercier P Roudier J Reviron D Functional categorization of HLA-DRB1 alleles in rheumatoid arthritis: the protective effect Hum Immunol 2003 64 930 935 10.1016/S0198-8859(03)00186-1 14522089

[B16] Tamiya G Shinya M Imanishi T Ikuta T Makino S Okamoto K Furugaki K Matsumoto T Mano S Ando S Whole genome association study of rheumatoid arthritis using 27 039 microsatellites Hum Mol Genet 2005 14 2305 2321 10.1093/hmg/ddi234 16000323

[B17] Barton A Thomson W Ke X Eyre S Hinks A Bowes J Gibbons L Plant D Wilson AG Marinou I Re-evaluation of putative rheumatoid arthritis susceptibility genes in the post-genome wide association study era and hypothesis of a key pathway underlying susceptibility Hum Mol Genet 2008 17 2274 2279 10.1093/hmg/ddn128 18434327 PMC2465799

[B18] Barton A Thomson W Ke X Eyre S Hinks A Bowes J Plant D Gibbons LJ Wilson AG Bax DE Rheumatoid arthritis susceptibility loci at chromosomes 10p15, 12q13 and 22q13 Nat Genet 2008 40 1156 1159 10.1038/ng.218 18794857 PMC2662493

[B19] Gregersen PK Silver J Winchester RJ The shared epitope hypothesis. An approach to understanding the molecular genetics of susceptibility to rheumatoid arthritis Arthritis Rheum 1987 30 1205 1213 10.1002/art.1780301102 2446635

[B20] Hinks A Worthington J Thomson W The association of PTPN22 with rheumatoid arthritis and juvenile idiopathic arthritis Rheumatology (Oxford) 2006 45 365 368 10.1093/rheumatology/kel005 16418195

[B21] Orozco G Hinks A Eyre S Ke X Gibbons LJ Bowes J Flynn E Martin P Wilson AG Bax DE Combined effects of three independent SNPs greatly increase the risk estimate for RA at 6q23 Hum Mol Genet 2009 18 2693 2699 10.1093/hmg/ddp193 19417005 PMC2701332

[B22] Stahl EA Raychaudhuri S Remmers EF Xie G Eyre S Thomson BP Li Y Kurreeman FA Zhernakova A Hinks A Genome-wide association study meta-analysis identifies seven new rheumatoid arthritis risk loci Nat Genet 2010 42 508 514 10.1038/ng.582 20453842 PMC4243840

[B23] Thomson W Barton A Ke X Eyre S Hinks A Bowes J Donn R Symmons D Hider S Bruce IN Rheumatoid arthritis association at 6q23 Nat Genet 2007 39 1431 1433 10.1038/ng.2007.32 17982455 PMC2674282

[B24] Orozco G Barton A Update on the genetic risk factors for rheumatoid arthritis Expert Rev Clin Immunol 2010 6 61 75 10.1586/eci.09.72 20383892

[B25] Nakashima H Miyake K Inoue Y Shimizu S Akahoshi M Tanaka Y Otsuka T Harada M Association between IL-4 genotype and IL-4 production in the Japanese population Genes Immun 2002 3 107 109 10.1038/sj.gene.6363830 11960309

[B26] Fishman D Faulds G Jeffery R Mohamed-Ali V Yudkin JS Humphries S Woo P The effect of novel polymorphisms in the interleukin-6 (IL-6) gene on IL-6 transcription and plasma IL-6 levels, and an association with systemic-onset juvenile chronic arthritis J Clin Invest 1998 102 1369 1376 10.1172/JCI2629 9769329 PMC508984

[B27] Dawson SJ Wiman B Hamsten A Green F Humphries S Henney AM The two allele sequences of a common polymorphism in the promoter of the plasminogen activator inhibitor-1 (PAI-1) gene respond differently to interleukin-1 in HepG2 cells J Biol Chem 1993 268 10739 10745 8388372

[B28] Lockett KL Hall MC Xu J Zheng SL Berwick M Chuang SC Clark PE Cramer SD Lohman K Hu JJ The ADPRT V762A genetic variant contributes to prostate cancer susceptibility and deficient enzyme function Cancer Res 2004 64 6344 6348 10.1158/0008-5472.CAN-04-0338 15342424

[B29] Turner DM Williams DM Sankaran D Lazarus M Sinnott PJ Hutchinson IV An investigation of polymorphism in the interleukin-10 gene promoter Eur J Immunogenet 1997 24 1 8 10.1111/j.1365-2370.1997.tb00001.x 9043871

[B30] Chen H Wilkins LM Aziz N Cannings C Wyllie DH Bingle C Rogus J Beck JD Offenbacher S Cork MJ Single nucleotide polymorphisms in the human interleukin-1B gene affect transcription according to haplotype context Hum Mol Genet 2006 15 519 529 10.1093/hmg/ddi469 16399797

[B31] Wen AQ Wang J Feng K Zhu PF Wang ZG Jiang JX Effects of haplotypes in the interleukin 1beta promoter on lipopolysaccharide-induced interleukin 1beta expression Shock 2006 26 25 30 10.1097/01.shk.0000223125.56888.c7 16783194

[B32] Hermans PW Hibberd ML Booy R Daramola O Hazelzet JA de Groot R Levin M 4G/5G promoter polymorphism in the plasminogen-activator-inhibitor-1 gene and outcome of meningococcal disease. Meningococcal Research Group Lancet 1999 354 556 560 10.1016/S0140-6736(99)02220-5 10470700

[B33] Correa PA Gomez LM Cadena J Anaya JM Autoimmunity and tuberculosis. Opposite association with TNF polymorphism J Rheumatol 2005 32 219 224 15693080

[B34] Hull J Ackerman H Isles K Usen S Pinder M Thomson A Kwiatkowski D Unusual haplotypic structure of IL8, a susceptibility locus for a common respiratory virus Am J Hum Genet 2001 69 413 419 10.1086/321291 11431705 PMC1235312

[B35] Rodriguez-Carreon AA Zuniga J Hernandez-Pacheco G Rodriguez-Perez JM Perez-Hernandez N Montes de Oca JV Cardiel MH Granados J Vargas-Alarcon G Tumor necrosis factor-alpha -308 promoter polymorphism contributes independently to HLA alleles in the severity of rheumatoid arthritis in Mexicans J Autoimmun 2005 24 63 68 10.1016/j.jaut.2004.11.002 15725578

[B36] van de Geijn FE Roos A de Man YA Laman JD de Groot CJ Daha MR Hazes JM Dolhain RJ Mannose-binding lectin levels during pregnancy: a longitudinal study Hum Reprod 2007 22 362 371 10.1093/humrep/del392 17099209

[B37] Tregouet DA Escolano S Tiret L Mallet A Golmard JL A new algorithm for haplotype-based association analysis: the Stochastic-EM algorithm Ann Hum Genet 2004 68 165 177 10.1046/j.1529-8817.2003.00085.x 15008795

[B38] Schaid DJ Rowland CM Tines DE Jacobson RM Poland GA Score tests for association between traits and haplotypes when linkage phase is ambiguous Am J Hum Genet 2002 70 425 434 10.1086/338688 11791212 PMC384917

[B39] QUANTO 1.1: A computer program for power and sample size calculations for genetic-epidemiology studies http://hydra.usc.edu/gxe

[B40] Klimiuk PA Sierakowski S Latosiewicz R Skowronski J Cylwik JP Cylwik B Chwiecko J Histological patterns of synovitis and serum chemokines in patients with rheumatoid arthritis J Rheumatol 2005 32 1666 1672 16142858

[B41] Lo SF Huang CM Lin HC Chen WC Tsai CH Tsai FJ Cytokine (IL-6) and chemokine (IL-8) gene polymorphisms among rheumatoid arthritis patients in Taiwan Clin Exp Rheumatol 2008 26 632 637 18799095

[B42] Khanna D Wu H Park G Gersuk V Gold RH Nepom GT Wong WK Sharp JT Reed EF Paulus HE Tsao BP Association of tumor necrosis factor alpha polymorphism, but not the shared epitope, with increased radiographic progression in a seropositive rheumatoid arthritis inception cohort Arthritis Rheum 2006 54 1105 1116 10.1002/art.21750 16572445

[B43] Balog A Klausz G Gal J Molnar T Nagy F Ocsovszky I Gyulai Z Mandi Y Investigation of the prognostic value of TNF-alpha gene polymorphism among patients treated with infliximab, and the effects of infliximab therapy on TNF-alpha production and apoptosis Pathobiology 2004 71 274 280 10.1159/000080062 15459487

[B44] Seitz M Wirthmuller U Moller B Villiger PM The -308 tumour necrosis factor-alpha gene polymorphism predicts therapeutic response to TNFalpha-blockers in rheumatoid arthritis and spondyloarthritis patients Rheumatology (Oxford) 2006 16720636 10.1093/rheumatology/kel175

[B45] Mugnier B Balandraud N Darque A Roudier C Roudier J Reviron D Polymorphism at position -308 of the tumor necrosis factor alpha gene influences outcome of infliximab therapy in rheumatoid arthritis Arthritis Rheum 2003 48 1849 1852 10.1002/art.11168 12847678

[B46] Bayley JP Ottenhoff TH Verweij CL Is there a future for TNF promoter polymorphisms? Genes Immun 2004 5 315 329 10.1038/sj.gene.6364055 14973548

[B47] Lindberg J Af Klint E Ulfgren AK Stark A Andersson T Nilsson P Klareskog L Lundeberg J Variability in synovial inflammation in rheumatoid arthritis investigated by microarray technology Arthritis Res Ther 2006 8 R47 10.1186/ar1903 16507157 PMC1526587

[B48] Martinez A Pascual M Pascual-Salcedo D Balsa A Martin J de la Concha EG Genetic polymorphisms in Spanish rheumatoid arthritis patients: an association and linkage study Genes Immun 2003 4 117 121 10.1038/sj.gene.6363931 12618859

[B49] Riyazi N Kurreeman FA Huizinga TW Dekker FW Stoeken-Rijsbergen G Kloppenburg M The role of interleukin 10 promoter polymorphisms in the susceptibility of distal interphalangeal osteoarthritis J Rheumatol 2005 32 1571 1575 16078336

[B50] Ates O Hatemi G Hamuryudan V Topal-Sarikaya A Tumor necrosis factor-alpha and interleukin-10 gene promoter polymorphisms in Turkish rheumatoid arthritis patients Clin Rheumatol 2008 27 1243 1248 10.1007/s10067-008-0893-1 18427872

[B51] Schluter B Raufhake C Erren M Schotte H Kipp F Rust S Van AH Assmann G Berendes E Effect of the interleukin-6 promoter polymorphism (-174 G/C) on the incidence and outcome of sepsis Crit Care Med 2002 30 32 37 10.1097/00003246-200201000-00005 11902285

[B52] Terry CF Loukaci V Green FR Cooperative influence of genetic polymorphisms on interleukin 6 transcriptional regulation J Biol Chem 2000 275 18138 18144 10.1074/jbc.M000379200 10747905

[B53] Hegedus CM Skibola CF Bracci P Holly EA Smith MT Screening the human serum proteome for genotype-phenotype associations: an analysis of the IL6 - 174G > C polymorphism Proteomics 2007 7 548 557 10.1002/pmic.200600366 17309100

[B54] Smeets TJ Barg EC Kraan MC Smith MD Breedveld FC Tak PP Analysis of the cell infiltrate and expression of proinflammatory cytokines and matrix metalloproteinases in arthroscopic synovial biopsies: comparison with synovial samples from patients with end stage, destructive rheumatoid arthritis Ann Rheum Dis 2003 62 635 638 10.1136/ard.62.7.635 12810425 PMC1754593

[B55] Kuuliala K Orpana A Leirisalo-Repo M Kautiainen H Hurme M Hannonen P Korpela M Mottonen T Paimela L Puolakka K Polymorphism at position +896 of the toll-like receptor 4 gene interferes with rapid response to treatment in rheumatoid arthritis Ann Rheum Dis 2006 65 1241 1243 10.1136/ard.2006.055137 16606645 PMC1798301

