# Supplementary material for: Polymorphisms in genes controlling inflammation and tissue repair in rheumatoid arthritis: a case control study
Source: BMC Med Genet. 2011 Mar 7;12:36. doi: 10.1186/1471-2350-12-36 (PMC3060109; doi:10.1186/1471-2350-12-36)
Supplement: Additional file 1 — Supplemental tables S1, S2, S3 and S4. Supplemental table S1. PCR primer sequences. Supplemental table S2. PCR conditions using 384 Tetrad PCR machine. Supplemental table S3. Touchdown protocols for PCR. Supplemental table S4. Primer sequences for SBE reactions. [file 1471-2350-12-36-S1.PDF]

Supplemental tables

Supplemental table 1. PCR primer sequences

Pool 1	Upper 5’→ 3’	Lower 5’ → 3’	rs number	change
NOS2A	GCAGGGCTAGGAGTAGGA	AGCCCCATATGTAAACCAA	rs2297518	608 S/L
PARP	CTGCCCCTGTTCTACCA	ACTGTAGGCCACCTCGAT	rs1136410	762V/A
**Pool 2**				
C1INH	CCTCCGCCATCTCTGT	GCTCGCCCTAACCTGA	rs4926	480 V/M
IL4	CTTGCCAAGGGCTTCCTTAT	TGGAAACTGTCCTGTCATGG	rs2243250	C-524T
**Pool 3**				
IL10	TCCCCTTACCTTCTACACAC	GACCCCTACCGTCTCTATTT	rs1800896	G-1082A
IL1B	CTTGCCCTTCCATGAAC	TGCCTCGAAGAGGTTTG	rs1143627	C-31T
TNFA.1	CCCCTCCCAGTTCTAGTT	GGGACACACAAGCATCA	rs361525	G-238A
			rs1800629	G-308A
			rs3093659	G-376A
TNFA.2	GGAGAATGTCCAGGGCTATG	AAAATCAGGGACCCCAGAGT	rs1800630	A-863C
			rs1799724	T-857C
**Pool 4**				
TLR4	ATGCCCCTACTCAATCTCTCT	GCCAGCCATTTTCAAGACT	rs4986790	299 D/G
			rs4986791	399 T/I
IL6	TTGTCAAGACATGCCAAGTGCT	GCCTCAGAGACATCTCCAGTCC	rs1800795	G-174C
IL8	AGCTTGCCTACTATAAATAACA	CTAGCCCTTGACCTCAG	rs2227306	C781T
**Pool 5**				
IL10	TCCCCTTACCTTCTACACAC	GACCCCTACCGTCTCTATTT	rs3021097	C-819T

Supplemental table 2. PCR conditions using 384 Tetrad PCR machine

Gene	Annealing temperature ^{o}C	Number of cycles	MgCl (mM)
TNFA (1)	60	35	1.5
TNFA (2)	60	35	1.5
IL1B	60	35	2.5
IL10	58	35	1
IL4	60	35	2
PAI1	60	37	1
C1INH	Touchdown 65>58	[a]	1
NOS	58	35	1.5
PARP	60	35	2
TLR4	58	35	1.5
IL8	Touchdown 63>56	[a]	1
IL6	58	35	1

a See Supplemental table 3.

Supplemental table 3. Touchdown protocols for PCR

Program	Touch down 65>58<br>Temperature ^{o}C	Time (minutes)	Program	Touch down 63>56<br>Temperature ^{o}C	Time (minutes)
Step 1	95	5:00	Step 1	95	5:00
Step 2	94	0:20	Step 2	94	0:20
Step 3	65 increment – 0.5/cycle	1:00	Step 3	63 increment – 0.5/cycle	1:00
Step 4	72	1:00	Step 4	72	1:00
Step 5	Go to step 2 additional 14x		Step 5	Go to step 2 additional 14x	
Step 6	94	0:20	Step 6	94	0:20
Step 7	58	1:00	Step 7	56	1:00
Step 8	72	1:00	Step 8	72	1:00
Step 9	Go to step 6 additional 19x		Step 9	Go to step 6 additional 19x	
Step 10	72	5:00	Step 10	72	5:00
Step 11	8	Forever	Step 11	8	Forever
Step 12	End		Step 12	End	

Supplemental table 4. Primer sequences for SBE reactions

	Primer sequence	primer length (bases)
**Pool 1**	5’ → 3’	
PrNOS2A_U	TTTTGCTCTTTCAGCATGAAGAGC	24
PrPARP_L	TTTTTTTTTTGCAGGTTGTCAAGCATTTCC	30
**Pool 2**		
PrIL4_U	TAAACTTGGGAGAACATTGT	20
PrC1INH_U	TTTTTTTTTTTTTTTTTTTTTTTTTTTTTTTTTTTTTTTTTTTTTTTGCAGCAGCCCTTCCT CTTC	66
**Pool 3**		
PrTNFA_2863U	AGTCGAGTATGTGGACCCCC	20
PrIL10_1082L	TTTTTTTTTTTTACCTATCCCTACTTCCCC	30
PrTNFA1238U	TTTTTTTTTTTTTTTTTTTTTTAGAAGACCCCCCTCGGAATC	42
PrIL1B_L	TTTTTTTTTTTTTTTTTTTTTTTTTTTTTTCTCCCTCGCTGTTTTTAT	48
PrTNFA_1308L	TTTTTTTTTTTTTTTTTTTTTTTTTTTTTTTTTTCTAGAGGCTGAACCCCGTCC	54
PrTNFA_1376U	TTTTTTTTTTTTTTTTTTTTTTTTTTTTTTTTTTTTTTTTTTTTTTTGCCTGCATCCTGTCT GGAA	66
PrTNFA_2857L	TTTTTTTTTTTTTTTTTTTTTTTTTTTTTTTTTTTTTTTTTTTTTTTTTTTTCTCTACATGGC CCTGTCTTC	72
**Pool 4**		
PrIL6_L	AATGTGACGTCCTTTAGCAT	24
PrIL8_L	TTTTTTTTTTCATAACTGACAACATTGAAC	30
PrTLR4_299U	TTTTTTTTTTTTTTTTTTTTTTTTTTTTTTTTTTTTTTTTCTTAGACTACTACCTCGATG	60
PrTLR4_399L	TTTTTTTTTTTTTTTTTTTTTTTTTTTTTTTTTTTTTTTTTTTTTTTTTTTTGATCTAAATAC TTTAGGCTG	72
**Pool 5**		
PrIL10_819U	TTTTTTTTTTTTTTTTTTTTTTTTTTTTTTTTTTTTTTTTTTTTTTTTTTTTTTTTTTTTTTCC CTTGTACAGGTGATGTAA	82
